# Host–Pathogen Interactions Influencing Zoonotic Spillover Potential and Transmission in Humans

**DOI:** 10.3390/v15030599

**Published:** 2023-02-22

**Authors:** Beatriz Escudero-Pérez, Alexandre Lalande, Cyrille Mathieu, Philip Lawrence

**Affiliations:** 1 WHO Collaborating Centre for Arbovirus and Haemorrhagic Fever Reference and Research, Bernhard Nocht Institute for Tropical Medicine, 20359 Hamburg, Germany; 2 German Center for Infection Research (DZIF), Partner Site Hamburg-Luebeck-Borstel-Reims, 38124 Braunschweig, Germany; 3 CIRI (Centre International de Recherche en Infectiologie), Team Neuro-Invasion, TROpism and VIRal Encephalitis, INSERM U1111, CNRS UMR5308, Université Claude Bernard Lyon 1, Ecole Normale Supérieure de Lyon, 69007 Lyon, France; 4 CONFLUENCE: Sciences et Humanités (EA 1598), Université Catholique de Lyon (UCLy), 69002 Lyon, France

**Keywords:** zoonosis, species barriers, Nipah virus, Ebola virus, innate immune antagonism factors, viral amyloidogenesis, interhuman transmission, viral spillover, Mobillivirus

## Abstract

Emerging infectious diseases of zoonotic origin are an ever-increasing public health risk and economic burden. The factors that determine if and when an animal virus is able to spill over into the human population with sufficient success to achieve ongoing transmission in humans are complex and dynamic. We are currently unable to fully predict which pathogens may appear in humans, where and with what impact. In this review, we highlight current knowledge of the key host–pathogen interactions known to influence zoonotic spillover potential and transmission in humans, with a particular focus on two important human viruses of zoonotic origin, the Nipah virus and the Ebola virus. Namely, key factors determining spillover potential include cellular and tissue tropism, as well as the virulence and pathogenic characteristics of the pathogen and the capacity of the pathogen to adapt and evolve within a novel host environment. We also detail our emerging understanding of the importance of steric hindrance of host cell factors by viral proteins using a “flytrap”-type mechanism of protein amyloidogenesis that could be crucial in developing future antiviral therapies against emerging pathogens. Finally, we discuss strategies to prepare for and to reduce the frequency of zoonotic spillover occurrences in order to minimize the risk of new outbreaks.

## 1. Introduction: Zoonotic Spillover of Animal Viruses to Humans

Approximately 75% of emerging infectious diseases in humans are zoonoses [1,2,3,4]. Over the last half-century, the incidence and diversity of many viral zoonotic infectious diseases appear to have increased, on occasion leading to notable epidemics or even pandemics with important consequences for health, travel and economies. Some obvious recent or historical examples include the flu [5], Ebola virus (EBOV) disease [6,7], Marburg virus (MARV) disease [8,9], acquired immune deficiency syndrome (AIDS) [10], Nipah virus (NiV) disease [11,12], Lassa fever [13,14], middle east respiratory syndrome (MERS) [15], severe acute respiratory syndrome coronavirus (SARS) [16,17] and more recently, the related virus SARS-CoV-2 causing the COVID-19 pandemic [18,19,20,21].

The zoonotic transmission of viral diseases is a complex and multifactorial phenomenon. There are many aspects that contribute to it, including the intrinsic characteristics and relationships between the host, the pathogen, their shared environment, the phylogenetic distance between host species, as well as potential inter-species interactions and environmental drivers such as changes in the ecology of the pathogen’s animal reservoir or vector species, amongst others [22,23]. However, emerging disease outbreaks all have in common that they are initiated by a single transmission or multiple spillover events followed by local human-to-human transmission that can on occasion become more widespread. Spillover can be defined as the cross-species transmission to humans of pathogens from wildlife [24]. In the opposite situation, the transfer of a pathogen from humans to wildlife is referred to as “spillback” [22]. Finally, the concept of “horizontal virus transfer” refers to the transmission of pathogens between different species, including organisms of different biological kingdoms [25]. In any spillover event, there will be a source host (that sheds the pathogen), a recipient host (that becomes infected by the pathogen) and in some instances, an intermediate host (that bridges pathogen transfer between species). In very general terms, the probability of triggering ongoing and sustained chains of transmission, leading to a potential epidemic, depends initially on the extent to which human populations are exposed to potentially disease-causing zoonotic agents. However, it would appear that by itself, the simple act of sharing a geographical area with infected reservoir species or vectors is insufficient, as the number of cases of transmission triggering efficient human-to-human spread is relatively rare, despite the fact that many people are in contact with wild animal species and despite the vast viral diversity found within wild animal species throughout the world from which spillover events can occur [4,23].

**Figure 1:**
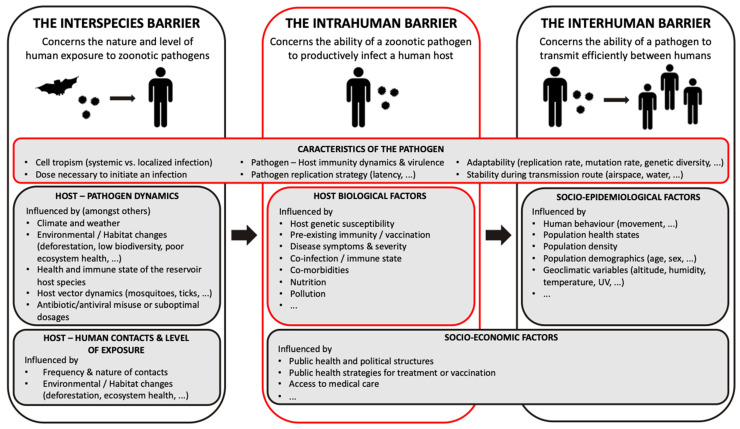
The three species barriers to zoonotic transmission and the factors that influence the ability of emerging or re-emerging zoonotic pathogens to cause epidemics/pandemics in humans. For a pathogen to be able to establish sustainable transmission chains in humans, all three barriers must be effectively surpassed. Boxes shown in red concern the pathogen and host factors and their interactions that are crucial in determining the relative success of a species jump for an animal virus to humans and which are developed further in this review. Further details are given in the text. Adapted with permission from [19]. Copyright 2022, Revue CONFLUENCE Sciences & Humanités.

Importantly, following transmission from an animal or vector host, many biological factors influence the relative ability or zoonotic potential of an animal virus to productively infect a human host and therefore the ability of a pathogen to replicate to sufficient levels to facilitate opportunities for ongoing transmission events in the new host species. At the biological level, the zoonotic potential of a virus, or indeed any non-human infectious pathogen, is dictated by the capacity of the pathogen to surmount a series of barriers to transmission and infection, known as the species barriers. These barriers can be divided into interspecies, intrahuman and interhuman barriers (Figure 1). The interspecies barrier determines the level and nature of exposure to zoonotic pathogens; the intrinsic, intrahuman barrier concerns the ability of the pathogen to survive a host immune response and thereafter to productively infect a human host; and finally, the human-to-human barrier reflects the ability of a pathogen to be transmitted efficiently between humans and therefore to be capable of establishing the transmission chains that can lead to outbreaks, epidemics or even pandemics [23,26,27]. Any zoonotic pathogen able to cause disease in humans is, to some extent, already able to more or less effectively overcome one or more of these barriers. For an animal pathogen to establish itself sustainably in humans through ongoing transmission chains, all three barriers must be surpassed.

On a pathogen–host cell level, these factors include the actual ability of the pathogen to replicate in the host, encompassing mechanisms governing cellular and tissue tropism, the virulence and pathogenic characteristics of the pathogen (influencing incubation periods, the onset of symptoms, transmission mechanisms and spread) and also the capacity of the pathogen to adapt and to evolve within the novel host environment, including when faced with host immune pressures. Many, if not all, of these host–pathogen interactions are also influenced by intrinsic host factors including genetic background, health-state, pre-existing disease/morbidity or immunity, as well as more distal socioeconomic factors (treatment and healthcare access, vaccination capacity and strategy, etc.) (Figure 1).

A constantly increasing number of viruses at high risk of cross-species spillover are found in bats, including very well-known human pathogens such as rabies, SARS-CoV-2, EBOV or NiV, just to cite a few. The Ebola and Nipah viruses are currently classified amongst the biosafety level 4 pathogens for which there are no or limited vaccines or treatments commercially available to prevent or cure infection. Of note, two licensed vaccines against EBOV are currently available for use in humans [28,29]. Whereas EBOV spread seems more restricted to primates including humans, NiV (and numerous other Henipaviruses) shows one of the broadest ranges of susceptible hosts to infection [30,31,32,33]. Both viruses have recently been placed in the WHO blueprint list of pathogens for which research and development of countermeasures should be prioritized in the face of their suspected high-risk pandemic potential [34]. However, their ability to spread between humans generally remains quite low, with an R0 of less than 1 compared to some other zoonotic viruses such as Influenza or SARS-CoV-2 viruses that regularly show an R0 higher than 5 [35]. For comparison, the Measles virus (MeV) is a reference among viruses in terms of high interhuman transmissibility with an R0 close to 18 on average, confirming its efficient adaptation to humans [36].

Understanding the main factors that promote pathogen transmission from wild animals and govern the ability of a pathogen to productively infect humans and allow ongoing spread is crucial to developing measures aimed at reducing the frequency of spillovers and epidemic/pandemic events as well as for our ability to contain or to manage them once they occur. This review aims to highlight the basic aspects of host–pathogen interactions that facilitate or contribute to spillover events and interhuman spread, with some specific examples from several recent and important human viruses of zoonotic origin, including NiV and EBOV. Some comparisons have been made with other, related viruses that are now almost exclusively human diseases, such as MeV and Respiratory Syncytial Virus (RSV). We also highlight the emerging understanding of the importance of the steric hindrance of host cell factors by viral proteins using a “flytrap”-type mechanism of viral protein amyloidogenesis that could be crucial in developing future antiviral therapies for emerging pathogens. Finally, we discuss strategies to prepare for and to reduce the frequency of zoonotic spillover occurrences to minimize the risk of new outbreaks.

## 2. First Steps: Contact, Infection and Amplification in the Host

Following transmission, the invading pathogen is amplified within the new host organism, either at the site of initial entry to the body, for example in the respiratory tract, or in peripheral tissues. During this amplification step, the ability of the pathogen to successfully replicate to high loads even in the presence of innate and/or adaptive immunity is thought to be one of the most important parameters that would then favor the efficient transmission of the pathogen from the infected individual to the next host.

At the cellular or tissue level, factors driving the amplification of the pathogen involve the molecular interplay between a pathogen’s immune antagonistic proteins and host cell factors and/or the ability of a pathogen to “hide” from the host immune system. To a greater or lesser extent, any pathogen that is able to infect and cause disease in humans has already evolved or acquired a range of mechanisms to overcome the host immune system. As mentioned above, these interactions are influenced in turn at the individual or host level by other drivers governing the immune response, including the host genetic background, pre-existing immunity or the presence of any infection or disruption to the normal functioning of the immune system including co-morbidity. Such drivers are in turn influenced on a larger scale at the community or country level by more distal socio-economic factors including public health strategies for treatment or vaccination or access to medical care.

In terms of onward transmissibility, what would appear important is the ability of the pathogen to replicate to high enough numbers at an ultimate shedding site within the infected individual. Pathogens that have acquired either some degree of or highly efficient transmissibility in humans through different mechanisms, including ‘airspace/aerosol’ or contact transmission, may present unique clues with which to understand this process when compared to related pathogens that are either much less or not transmittable via the same infection route. In this sense, some of the important specific drivers for pathogen amplification within the host are discussed below in terms of their potential influence on viral replication dynamics, pathogenesis mechanisms and transmissibility.

### 2.1. Viral Entry and Tissue Tropism

Part of the cross-species spillover potential of an animal virus notably relies on the high conservation of the virus entry receptors between species. Indeed, viruses harboring the broadest panel of hosts generally target receptors involved in very conserved essential functions. For example, the Hendra and Nipah viruses preferentially use the Ephrin B2 ligand as their main entry receptor [37,38]. Such a receptor is highly conserved among all mammals from bats to humans [39], since it is involved in embryogenesis, vasculogenesis and neurogenesis [40]. Whereas the success of an infection does not only rely on entry, such conservation at least avoids exclusion from this first step of the infection cycle. Of note, other related henipaviruses, including those found in bats in Africa, also appear able to use the Ephrin family of proteins as attachment/entry receptors, at least in in cellulo studies with recombinant proteins or surrogate virus entry systems [41,42,43,44,45].

Numerous Influenza viruses use different sialic acids depending on the host. The viral strains most often observed in humans generally originate from birds after an adaptation occurring in intermediate hosts such as pigs, which harbor both bird- and human-specific sialic acids. The ability of avian Influenza viruses to infect humans is thus linked to their potential to switch from the use of “avian-type” receptors to “human-type” airway sialic acids rather than the use of highly conserved common receptors between species [46,47,48,49,50,51]. The adaptation of avian viruses to mammalian hosts also often involves an adaptation of the viral polymerase to improve efficiency in mammalian cells [46].

In some cases, the use of host-restricted receptors seems to limit the range of hosts attained by the virus. EBOV uses T cell immunoglobulin mucin domain-1 (TIM-1) as an attachment factor [52], amongst others, thanks to interactions with phosphatidylserine present on the surface of the virus. Compared to Ephrin and sialic acids, this receptor is less conserved between species, which may partially explain the relative infection restriction to primates. Similarly, wildtype MeV only uses human CD150 (SLAMF1) and Nectin-4 as entry receptors which thus play an important role in the virus’ restriction to primates. Numerous other Morbilliviruses such as canine distemper virus (CDV), Phocine distemper virus or cetacean morbillivirus seem able to use more conserved equivalent receptors, known as Dog SLAM or Dog Nectin-4, shared between multiple species [53,54]. All three morbilliviruses seem able to spillover from their natural hosts (dogs, cats, cetaceans) to common hosts such as phocines (seals) with a high risk of recombination. It is of concern that some of these viruses closely related to CDV would seem thus able to infect and cause disease in non-human primates, suggesting that the risk of spillover to a broader spectrum of hosts is in fact quite high. Indeed, for CDV, only one mutation at position 540 of the viral surface H protein is required to allow the virus to use human SLAM, opening the possibility for the virus to at least enter human cells [53].

Following infection, a major factor that would appear to be important in determining whether or not a given pathogen is able to be efficiently and regularly transmitted from one human host to another is the specific tissue or organ site at which the pathogen is replicating and therefore amplified. Most pathogens have an affinity for specific tissues determined by the selective susceptibility of cells to a particular virus, its tropism and host defense mechanisms as well as local environmental factors including tissue barriers, temperature and pH. For some pathogens, this site corresponds to the same site as that of initial entry into the host organism, whereas other pathogens are known to have either distinct secondary sites of infection/amplification or are able to replicate on both a local and a systemic level. This secondary site may or may not also correspond directly to the relevant site of exit within the shedding individual, to which any successfully transmitted pathogen must ultimately attain.

At the individual level, drivers of infection and the transmissibility of certain pathogens would also appear to be closely linked to disease progression and severity as well as to the incubation period and onset of symptoms, although it is difficult to establish clear correlations or conclusions between different pathogens. For example, for MeV recent studies have highlighted the importance of tissue-specific temporal patterns of virus spread and resulting pathologies [55]. Such a model relies on the ability of MeV firstly to use CD150 to enter immune and lymphoid tissues following transmission and then, in the latter stages of infection, the Nectin-4 receptor hidden at the basolateral side of epithelial cells to invade epithelia, from which the virus will then be excreted and spread, notably by coughing. However, this model, which is very efficient for classical infection by MeV, loses its powerfulness in terms of transmission when the virus infects the brain, currently considered as a dead-end for infection. Likewise, for Influenza, studies have suggested that peak excreted titers and the time required for titers to reach a minimal infectious dose are critical phenotypes influencing transmissibility [56]. Both points are linked to the virulence and pathogenesis mechanisms of a pathogen and therefore are governed to some extent by the steps controlling the amplification of the pathogen within a newly infected host as well as by specific tissue tropism and damage either as a direct result of viral replication or as a consequence of a host immune response.

Both MeV and the closely related human paramyxovirus RSV are examples of once-zoonotic viruses that have evolved to become exclusively diseases of primates [55,57] and that have acquired different levels of efficiency in terms of their human-to-human transmissibility [58,59]. From this perspective, it is interesting to compare MeV and RSV and the highly pathogenic, re-emerging NiV, for which transmissibility among humans appears to be much less efficient. NiV is a highly pathogenic member of the genus Henipavirus within the family *Paramyxoviridae* that first emerged from fruit bats in Malaysia and Singapore in 1999 during an outbreak of severe respiratory disease in pigs and fatal encephalitis in humans [30,32,60,61]. Since 2001, smaller outbreaks of NiV infections in India and Bangladesh have regularly occurred, with reports of human-to-human transmission being an important hallmark in many of these later outbreaks [11,62,63,64]. MeV remains an important human virus causing many deaths in children annually. Although the World Initiative project of the eradication of measles came close to being successful with fewer than 8000 deaths/year in 2008, case numbers have continuously increased every year for the last 15 years due to a lower propensity of people being vaccinated. More than 200,000 deaths were reported in 2019, and these numbers may dramatically increase during the next few years mainly due to a severe loss of public confidence in vaccines, due mainly to unsubstantiated rumors and falsified data promoted on social media, and also since numerous countries have stopped their vaccination programs due to the COVID-19 pandemic. Indeed, worldwide, about 60% of vaccination campaigns were already either postponed or canceled by May 2020 [65]. RSV is one of the most common respiratory diseases in infants and young children worldwide [66]. The clinical signs of RSV bronchiolitis in children include wheezing, a cough and increased difficulty in breathing caused by infection of the bronchiolar airways. The nasal phase of an infection may cause irritation and nerve activation associated with sneezing, congestion and apnea in children and in animal models [67]. The major target of RSV is airway epithelial cells. In the lower respiratory tract, RSV infects bronchiolar and alveolar epithelial cells, whereas ex vivo experiments have shown efficient infection of the nasal epithelium [68,69,70].

All three of the viral infections caused by MeV, NiV and RSV are thought to originate in the airways after infection through the oronasopharyngeal route. From here, both MeV and NiV are able to spread from their initial infection of immune cells to associated lymphoid organs and the bloodstream before establishing systemic infection of other tissues, including the submucosa, tongue, buccal mucosa, trachea, nose and skin for MeV and endothelial cells in many organs for NiV, including notably the lung, kidneys and the central nervous system (CNS) [32,71]. MeV initially infects antigen-presenting cells (APCs) in the respiratory tract, which will then transmit the infection to activated lymphocytes, thus disseminating the virus in cis in blood circulation and then by contact with lung epithelial cells in the respiratory tract for the shedding of the virus [72,73]. In comparison, NiV seems able to infect a subpopulation of alveolar APCs; these cells will then transmit the virus in cis to lymphocytes thanks to heparan sulfate binding but without infecting them. Circulating immune cells loaded with viral infectious particles on their surface can then enter the blood and lymphatic circulatory systems to trans-infect endothelial cells before further spreading to organ parenchyma [74,75]. In contrast, the site of amplification for RSV infection is thought to remain the epithelial cells of the upper (nasal) and lower respiratory tract [68].

Following the local infection of respiratory tract DCs and alveolar macrophages with MeV, the virus spreads to the bronchus-associated lymphoid tissue (BALT) with the peak of viral infection associated with the presence of syncytia in bronchial lymphoid tissue 7–9 days post-infection (for detailed reviews see [55,76]). At this stage, systemic MeV-infected immune cells are able to contact and/or infiltrate epithelia leading to the infection of respiratory tract epithelial cells. Studies have revealed that multiple subtypes of epithelia may become infected with different levels of resulting tissue damage, including the infection of the upper respiratory tract and epithelial cells of the nasal cavity. Notably, it has been postulated that for MeV, this infection of nasal tissue may be an important driver behind the efficient host-to-host human transmission of this pathogen [55]. The infection of columnar epithelial cells in the trachea and bronchi leads to the exfoliation of these cells into the respiratory tract and may also contribute to pathogen shedding. In support of these observations, the use of a human airway epithelia model infected with MeV showed that masses of infected cells with high amounts of cell-associated virus dislodge from the epithelial surface. Epithelial function seems to be preserved during infection, and these “infectious centers” remain ‘alive’ and retain their metabolic activity. Additionally, they were as efficient as a cell-free virus to infect macrophages by cell–cell contact [77]. Accordingly, the authors propose that this mechanism may be an important factor explaining the high R0 of MeV, in that infectious centers would be expelled by coughing and sneezing, thereby promoting longer survival of the virus in the environment and the infection of the next host with a high infectious dose. At the level of virus amplification in host epithelia, lesions are observed in the latter stages of disease as well as the extensive infiltration of infected tissue by immune cells. It is currently unclear to what extent the host immune response may also contribute to the formation of these pathological lesions and therefore to what extent the host response may contribute to the transmissibility of the virus.

In contrast to MeV, RSV infection of the human airway epithelium is not thought to cause direct damage to the epithelium but rather the induction of proinflammatory chemokine and cytokine responses, often correlating with disease severity, that then attract and activate immune cells including neutrophils, monocytes, macrophages and T cells with resulting airway epithelium damage [68,78]. Importantly, the transmission of RSV is thought to occur mostly through droplet or contact transmission and is likely, not airborne in the same way as MeV despite similar respiratory symptomatology. Understanding the mechanisms underlying such differences and transmission potential, especially for an emerging viral disease, is obviously important in predicting the ability and efficiency of a pathogen to spread within the human population.

An important characteristic of NiV is its wide cellular and host tropism. Its receptor, Ephrin B2/B3, is expressed on leukocytes that have been shown to disseminate NiV via blood to many tissues. In fact, Ephrin B2/B3 is expressed in almost all tissues in the body, including the central nervous system (CNS), which contributes to the virus’ wide tropism in terms of hosts and tissues. Although encephalitis and lesions of the CNS are primarily responsible for fatal clinical disease in humans, pigs show mainly respiratory symptoms and in some cases neurological signs [79,80]. Such observations have been reproduced in experimentally infected pigs with the highest virus content in the upper and lower respiratory tract [81,82]. Studies of NiV infection in a hamster model showed that the respiratory epithelium is the initial area for NiV replication in endothelial cells that then leads to viraemia and the systemic spread of infection [83,84]. Immunohistological data of in vivo infection demonstrate that NiV infects epithelial cells at mucosal surfaces, including the respiratory tract, the kidneys and the bladder, consistent with viral shedding in airway secretions and urine [32,85,86]. In human cases of NiV infection, respiratory symptoms may vary but many reports have suggested an important role of respiratory secretions in human-to-human transmission of NiV [87].

Virus–receptor interaction is crucial in regulating viral tropism and pathogenesis. The fact that certain viruses infect and replicate in a wide range of cells enhances their high pathogenicity. This is also the case for EBOV, which replicates in various tissues and cell types due to the ability of its glycoprotein GP to interact with multiple attachment factors on many cellular surfaces and the presence of the Niemann Pick C1 (NPC1) receptor, used by EBOV, on a wide range of cell types and tissues [88].

The ability of a pathogen to infect a specific cell type, organ or host may also vary over the course of the disease; however, it is clear that although wide host tropism can impact disease transmission, cellular and organ tropism can mostly affect the severity of the disease. From this point of view, understanding a pathogen’s tropism dynamics may help to limit disease and spread by developing specific treatments that reduce a pathogen’s access to the targeted cells.

### 2.2. Virulence and Pathogenesis

As outlined above, once the transmitted pathogen finds an entry into the human host, its interaction with the host immune system, as well as the impact of any other underlying medical conditions, ultimately determines whether infection is established and whether it progresses to clinical disease. Virulence is one of a number of possible outcomes of host–pathogen interactions, involving a series of complex and dynamic phenomena between both host and viral factors [89]. The virulence of a pathogen depends on several characteristics of the pathogen itself and its replication in the host, including its relative “aggressiveness” and “toxicity”. Aggressiveness can be defined as the ability of a microorganism to invade and survive in the tissue of a host by inhibiting or destroying the defense mechanisms of the host. Pathogenesis is attributed to the capacity of a microorganism to damage host tissue through replication or the release of toxins and/or toxic metabolic substances, often as a direct result of replication, or as a consequence of activation and infiltration of immune cells. Indeed, the idea of pathogenesis encompasses the steps of viral dissemination and amplification within the host organism from the initial portal of entry through local replication and spread to target organs and/or sites of shedding into the environment and the ability of a pathogen to cause disease in the infected host.

Drivers potentially influencing onward transmissibility also, therefore, include the virulence characteristics of the infecting pathogen. As detailed above, the infecting pathogen must be able to overcome the inhibitory effects of host defenses and cellular susceptibilities to infection. Virulence characteristics allow the virus to initiate infection, spread in the body and replicate to large enough numbers to then favor transmission. Some pathogens have developed genotypes that have highly effective virulence including more efficient transmission. Although this would appear to sometimes be the case with bacteria [90,91], the same does not always hold true for viruses, for which many highly virulent virus families or strains are not associated with higher transmissibility [92]. On the contrary, the high pathogenicity of a viral pathogen, linked to high fatality rates in humans, may lead to limited transmissibility, especially if the host succumbs to disease before transmitting the pathogen. Some human viral diseases, such as EBOV, however, appear to continue to be highly transmissible through close contact transmission even after the death of the disease victim, often through traditional funeral and burial practices [93,94]. Other important factors include the ability of the pathogen to replicate under specific conditions including inflammation and a host febrile response and in the presence of host inhibitory factors and interferon.

### 2.3. Viral Non-Structural Proteins, Amyloidogenesis and Antiviral Response Antagonism

Antagonizing host immune responses is key for the success of viral infection and transmissibility, and notably relies on specific viral proteins, including many that are now thought to employ mechanisms related to amyloidogenesis that remain to be fully delineated. Among viral strategies to overcome host defense mechanisms, the use of specialized viral proteins is especially striking for zoonotic pathogens. In paramyxoviruses in particular, the non-structural C, V and W proteins act as virulence factors counteracting the innate immune response [95,96,97,98].

So far, an under-investigated aspect of these viral proteins’ biology is their ability to undergo phase separation and the link with host cell function interference. As a matter of fact, such an ability could be key in potentiating spillover in humans. Intrinsically disordered regions in proteins are defined as those devoid of stable secondary and tertiary structures [99] and are at the origin of biomolecular condensates through various phenomena of phase separation. Liquid–liquid phase separation consists of the segregation of groups of molecules resulting in the de-mixing of the cellular content into distinct liquid phases with different solute concentrations and the formation of droplets, rather like oil in water. In cells, liquid–liquid phase separation notably drives the formation of membraneless organelles such as centrioles, the nucleolus, stress granules or Cajal bodies. Liquid droplets can further undergo gelation and liquid-to-solid phase transition producing jellified or solid biomolecular condensates [100,101,102].

The maturation and nucleation of the condensates formed via liquid–liquid phase separation can give rise to fibrils with amyloidogenic properties [103,104]. Despite the extensive literature regarding phase separation [100], the study of virus-derived amyloid-like fibrils and their role in pathogenesis is still in its infancy [102,105,106,107]. Attention to this field has notably increased in the last couple of years with the demonstration of amyloidogenesis by the Spike, nucleoprotein and envelope proteins of SARS-CoV-2 and their possible involvement in pathogenesis [108,109,110,111].

A striking example of an amyloidogenic viral protein was recently reported both in vitro and in vivo in the case of the Rift Valley fever virus (RVFV) for the viral NSs protein [112]. The NSs protein is the main RVFV virulence factor [113] and forms filamentous structures in the nuclei and cytosol of infected cells [114]. These filaments were proven to meet the criteria of amyloids [115]: they are formed by the assembly of many straight unbranched NSs fibrils (fibrillar aggregates), they are stained by amyloid-binding dye Thioflavin-S, they grow in an amyloid-type fashion with an increase in size rather than in a number of filaments and they are resistant to strong detergents [112]. Abrogating NSs fibrillation results in the loss of virus-mediated suppression of an IFN response. A scenario where the fibrillar form of the viral protein serves as a trap for host proteins is attractive and is, for example, supported by the NSs-mediated degradation of key IFN response factor protein kinase R [116] and the NSs filament-mediated sequestration of host transcription factors [117]. RVFV is an emerging zoonotic mosquito-borne pathogen that can infect livestock and humans, causing hepatitis and encephalitis in severe cases [118]. Very little is known about RVFV replication in mosquitoes and how the insect vector deals with infection [119,120]. Interestingly, NSs filaments are either not observed or rapidly cleared in some mosquito cell lines infected with RVFV [112]. The ability of NSs to fibrillate without apparent restriction in vertebrates, as opposed to what happens in arthropod cells where NSs expression and filamentation appear to be somehow controlled, may contribute to the difference in pathogenesis between mosquitoes and humans. Indeed, RVFV causes presumably non-cytopathic persistent infection in mosquitoes with little detrimental effects, whereas cytopathicity is observed in vertebrate cells [112,120,121].

**Figure 2:**
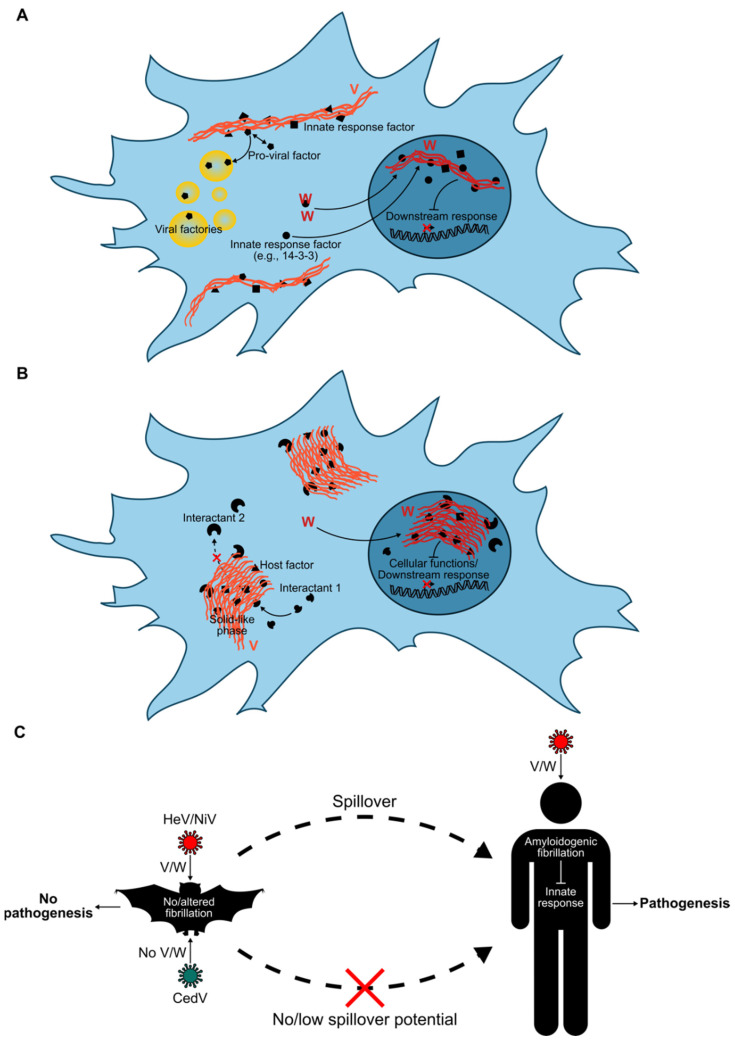
Involvement of viral non-structural protein amyloidogenesis in immune response blockage and spillover potential. (**A**) As an example of fibrillation-prone viral proteins interfering with host cell functions, Henipavirus V and W are proposed to form fibrillar filaments with properties of amyloids, either in the cytosol (for V) or the nucleus (for W) of non-bat cells, as a virulence strategy to prevent and counteract the innate immune response by acting like a “flytrap”, sequestering key cell effectors. The filaments could also be useful for the virus to recruit cellular pro-viral factors and target them to intracellular viral factories for example. (**B**) These filaments may further evolve into large solid-like condensates resulting in steric hindrance in the host cell, thereby trapping innate immune interactors without the need for very specific and strong interactions. In this scenario, steric hindrance would hamper the downstream actions of the innate effectors. (**C**) Differential ability of amyloid fibrillation by V and W between the bat reservoir and humans could be at the origin of the absence or development of pathogenesis and may be a determinant of the spillover potential of the viruses. Further details and references are provided in the text.

Paramyxoviruses also use intrinsic disorder to their advantage [105]. Similarly to mosquito-borne viruses, bat-borne viruses with zoonotic potential, such as Henipaviruses, including Hendra virus (HeV) and NiV, express virulence factors that play a key role in the evasion of the host antiviral response [98,122,123]. In particular, the non-structural V and W proteins of HeV and NiV are encoded by the P gene via the co-transcriptional editing of P mRNA [63,122]. They antagonize the type I interferon (IFN-I)-mediated and chemokine/proinflammatory response by targeting a myriad of signal transducers in these pathways such as innate immune sensor MDA5 [124], STAT [125] or 14-3-3 proteins [126,127]. V and W were shown to be enriched in intrinsically disordered regions and to form amyloid-like fibrils in vitro [105,128,129]. The staining of transfected and infected mammalian cells with the amyloid-binding dye Congo red suggests that fibrils also form in cellulo [128,129]. On the basis of the well-known structure-function paradigm, establishing a link between these peculiar structures and the functional role of V and W is appealing (Figure 2A,B).

Mechanistically, how Henipaviruses antagonize host innate immune response is not yet fully understood. V and W bind STAT1, STAT2 and STAT4 [130]. V inhibits STAT1 nuclear translocation and promotes its degradation by binding a component of the ubiquitin ligase E3 complex [131,132,133], whereas W sequesters the cellular protein in an inactive form in the nucleus [125,131]. V binds pattern recognition receptors MDA5 and LGP2 as well as MDA5 signaling regulator PLK1 to prevent the detection of viral double-stranded (ds)RNA and thus downstream IFN-I induction [124,134]. NiV V and W can also block IFN-β induction by targeting IKKɛ and dsRNA sensor TLR3 pathways. NiV W inhibits TLR3 signaling and downstream IFN-β induction by mediating the loss of active IFN regulatory factor 3 (IRF3), potentially by targeting its phosphorylated form or a nuclear factor involved in IRF3 activation [135]. V and W also inhibit the production of chemokines and modulate an inflammatory response in vivo [136]. NiV W binds to and hijacks 14-3-3 proteins, sequestering them in the nucleus and promoting 14-3-3-mediated nuclear export of p65, resulting in the inhibition of an NF-κB-induced proinflammatory response and cytokine production [126,127]. V and W are therefore multifunctional proteins and their ability to fibrillate could be a way to trap and sequester host proteins involved in innate and inflammatory responses. In other words, the formation of fibrillar structures with amyloidogenic properties may act as a “flytrap” for host proteins, allowing the targeting of a broad range of cellular partners. It can be hypothesized that the interaction would not necessarily need to be very strong since large filamentous structures may be efficient in capturing many host proteins, as a “cast-a-wide-net” strategy (Figure 2A).

These structures that may evolve into solid phases could thus both spatially and sterically impede host proteins, preventing their interaction with one another and also cellular trafficking for example, with the ultimate goal of disturbing the proper functioning of the innate response (Figure 2B). In the intracellular environment, steric and spatial hindrances in general, resulting from the formation of these fibrillar structures, could indirectly limit the action of cellular factors by restraining their motion and thus their access to their targets. Consequently, this non-specific strategy of indirect interference with host cell function by crowding the space may retain some efficiency irrespective of the species, even if strong interactions do not exist. On the other hand, filaments could also serve as platforms to recruit pro-viral host factors and, for example, to target them to intracellular viral factories (Figure 2A).

Henipavirus infection in bat cells is poorly investigated and thus only a few data are available, but some speculations can be made based on what is known on bat biology. The ability of bats to mount an efficient IFN-I-mediate innate immune response is thought to be at the origin of their unique property to carry (possibly persistently) many zoonotic viruses without developing disease [137,138,139,140]. Despite a lack of knowledge regarding bat immunology, shared immune features with other mammals and significant variations, which may relate to the singular properties of bats, have been reported [32,33,139]. Firstly, bats are the only mammals able to freely fly. Flying involves a high metabolic cost, and it is speculated that daily cycles of flight-induced elevation of metabolic rates and concomitant elevated body temperatures stimulate immune system activation resulting in an absence of disease onset [141]. Incidentally, adaptation to flight likely demanded an accommodation to the harmful side effects of high metabolic rates and is thus proposed to have influenced genetic changes in the bat immune system [142].

Regarding viral infection in bats, flight-related increased metabolism and body temperature could have distinct effects. Body temperature during flying can reach values ranging from 38 to 41 °C, which mimics the typical fever temperatures observed during the febrile state in humans over the course of a viral disease. Such temperatures are known to potentiate innate immune responses [141]. One can easily hypothesize that chronically increased body temperature may antagonize the action of the viral non-structural proteins such as V and W and their fibrillation by several means that are not mutually exclusive and that might depend on the action of the innate immune system or not. For example, V and W expression, synthesis and degradation could be temperature dependent; higher temperatures may thus result in an absence or insufficient amount of V/W to enable fibrillation. It may also render V and W unable to fibrillate or promote the degradation of V/W amyloid-like fibrils. The expression of temperature-related proteins, namely, heat shock proteins (HSPs), whose basal levels are higher in bats compared to other mammals [143], might drive the inhibition of fibrillation by altering the 3D conformation of V and W proteins, either at the monomer or fibril level. Of note, NiV uses the cellular protein HSP90 for chaperone-dependent folding of its L polymerase [144], and the high expression of this chaperon and others in bats [143] may promote non-pathologic persistent infection by favoring virus replication via interaction with the L protein while restraining the action of virulence factors. Whether V/W fibrillation happens in bats thus warrants investigation, since bat peculiarities are expected to influence the physico-chemical properties of proteins at the cellular level. As temperature and intracellular environment, notably, encompassing metabolic processes and enzymatic reactions agitating the intracellular compartment mechanically and by heat dissipation, are crucial factors determining both molecular motion in cells and protein–protein interactions [145,146,147], it is possible that the interaction profile of V/W and/or of V/W amyloid-like fibrils with host proteins differs in bat cells. That is, the increased mobility of intracellular molecules may promote the disruption of host protein–viral amyloid interactions either preventing interaction and/or favoring the interaction of viral amyloids with host proteins differently from that seen in human cells. As a result, the deleterious effects of viral amyloids would be restrained, allowing better control of infection (Figure 2C).

The critical role of V/W in enabling henipaviruses to cross the species barrier and spill over into humans is further supported by two major observations. Cedar virus, another Henipavirus closely related to HeV and NiV, but that is non-pathogenic in animal models that are usually highly susceptible to Henipavirus infection, is devoid of these proteins and induces a much stronger IFN response than either HeV or NiV following infection [148,149,150]. Incidentally, Cedar virus has been found in no other species than bats and would appear to lack zoonotic spillover potential (Figure 2C). Secondly, HeV and NiV edit their P mRNA with a remarkably high frequency in order to produce high levels of V and W proteins, a peculiarity that may be tied to their high pathogenicity [151,152]. In addition, the recent emergence of a newly identified zoonotic Henipavirus, Langya virus, which appears able to cause febrile illness in humans, may further corroborate this assumption, since its genome seems to encode both V and W [153,154].

Building on all of these data, it is tempting to speculate that the utilization of viral protein fibrillation at the expense of the host cell may be extended to other viruses. The VP35 protein of EBOV, which, like some other members of the filovirus family, is also suspected to be a bat-borne virus, interferes with the integrated cellular stress response by co-aggregating with and sequestering stress granule proteins within viral inclusion bodies, thereby inhibiting stress granule assembly [155,156]. Given the role of VP35 in blocking an IFN response [157], it is conceivable that VP35 antagonizes an innate antiviral response using a similar strategy. Of further note, the murine cytomegalovirus M45 protein is also amyloidogenic and forms heteromeric amyloid fibrils with host proteins, a process blocking the necroptosis of infected cells [158]. Altogether, these examples point to a functional role of fibrillar aggregate formation and liquid–liquid phase separation by viral proteins, with the former possibly being of a solid-like nature as a result of the maturation of liquid-like condensates (Figure 2A,B).

## 3. Pathogen Genetics and Potential for Adaptation

Virulence can be affected by changes in pathogen population genetics at the consensus level but also by the genetic variability of the pathogen population as a whole. This is certainly true for RNA viruses in particular that exist as a population of closely related genetic variants within the host setting. Indeed, the ability of a pathogen to quickly adapt and to generate a genetically diverse population is considered critical for both the survival of a pathogen faced with a range of selective pressures and its capacity for ongoing transmission [159,160]. Indeed, for a zoonotic virus, successful replication and transmission in a novel host species are likely accompanied by the selection of viral genotypes/phenotypes that impact processes including receptor binding, entry and fusion, interactions with host proteins required for viral replication and/or escape from restriction factors and the modulation of immune responses.

In this sense, factors enabling viral pathogens to acquire novel traits through high mutation rates and/or a propensity to acquire novel genetic material through re-assortment or recombination are important drivers of potential for increased pathogen replication, amplification, adaptation and transmission within the host. Studies with polioviruses, for example, have demonstrated that high-fidelity mutants, i.e., producing viral populations with little genetic diversity/variability, are attenuated in mice, and this is despite apparent overall identical consensus sequences when compared with wild-type strains [161,162,163].

Opportunities for pathogen evolution/adaptation and change can also be considered as potential drivers influencing onward transmissibility. In this respect, patterns of increased transmissibility may also potentially involve an emerging pathogen already equipped for somewhat limited human-to-human transmission or a mutated pathogen expressing a trait that conveys increased disease severity and/or an increase in transmission. In this sense, it is probably true that drivers that are associated with emerging infectious diseases, and spillover events from animal reservoirs [23,164] are often to some extent also relevant for the “emergence” of pathogens displaying efficient interhuman transmissibility. Indeed, following zoonotic transmission to a new host species, the emergence of viral mutations and their selection in the novel host environment likely lead to a gradual optimization of the interactions between the virus and its new host, or more precisely, between the viral proteins and host cells [165,166].

During replication, changes will occur in viral genomes, often affecting promoter regions and/or protein-coding sequences as a result of fidelity-linked replication mechanisms involving the viral polymerase/replication machinery and its cofactors and replication rates. If receptor distribution and expression are similar between the reservoir and the novel recipient host, i.e., humans, changes are not generally seen in terms of actual disease patterns, but the optimization of the virus to a new host can and does give rise to the appearance of new viral variants within a population, particularly in the context of ongoing interhuman transmission, such as that seen with larger-scale epidemics/pandemics. In this situation, the virus is given ample opportunity to adapt and evolve under the selective pressure of host immune responses at both the individual and population levels, as well as during extracellular transmission events. Such changes will often affect interactions between viral surface proteins and their cellular receptors or between viral replication enzymes and their required cellular cofactors or partners [46,47,165,167,168,169].

However, selection and virus adaptation or “optimization” can occur at many levels; variants may show increased or prolonged shedding owing to higher replication levels or rates, thus increasing the chances of spread to susceptible individuals. Likewise, variants might show better survival in the environment, i.e., during airspace transmission in droplets, require a lower dose of virus to initiate productive infection in a newly infected individual, show an increased affinity for human cell receptors or be better at evading innate or acquired immunity through antagonistic mechanisms or through selected variations in surface epitopes. Such changes have been commonly observed during zoonotic outbreaks in humans, including during the 2013–2016 outbreak of EBOV in West Africa and again with the emergence of novel, more transmissible “variants of concern” during the current COVID-19 pandemic, including the “delta” or “omicron” variants and subvariants [170,171,172,173].

Indeed, EBOV is a good example of a virus for which adaptation mutations appear to be relevant in terms of virus infection, evolution and ability to spread in humans following a species jump. EBOV replication results in a quasispecies of variant genomes displaying variable fitness. As for many RNA viruses, EBOV encodes an RNA-dependent RNA polymerase (RdRp) lacking proofreading ability and is thus prone to generate errors during replication that can result in higher mutation rates [174]. Although the EBOV mutation rate depends on many factors, such as population size and demographic factors (bottlenecks, growth of the population, etc.) and cannot be fully established, in humans, this rate is estimated to be around ∼4.7 × 10^−4^ substitutions/site/year on average from all outbreaks [175]. During the 2013–2016 EBOV pandemic, several fixed mutations were identified, at least two of which were under positive selection and showed increased fitness but no obvious differences in pathogenicity in humans when compared to the original wild-type virus [167,168,176]. However, some of these mutations, located in the EBOV glycoprotein (GP), were shown to have an impact on EBOV infectivity in vitro. For example, a mutation in residue 544 affects GP stability and fusion capacity, whereas a mutation in residue 82 affects GP proteolytic cleavage. Importantly, the combination of these mutations increases the kinetics of GP fusion and EBOV infectivity in vitro [177].

In additional in vitro studies, changes in mutation rates were identified with the serial passaging of EBOV in both human and bat cell lines. Whereas mutation rates were similar in both cell lines, the authors showed that bat cell lines are more susceptible to generating EBOV variants with a high number of mutations in the mucin-like domain of the GP. Mutants from both cell lines were shown to have a replicative advantage over the original wild-type virus [178]. In the case of animals, it was shown that the same single substitutions in the EBOV polymerase (L), nucleoprotein (NP) or GP that occurred during the 2013–2016 EBOV outbreak have an impact on EBOV pathogenicity. Whereas the single mutations in NP and L seemed to decrease the virulence of the virus in mice and ferrets, the GP mutant increased virulence [179]. Other studies have shown that consecutive passaging of EBOV in animals influences its infectivity and virulence in the host species used and also in new host species [180,181,182]. Altogether, these studies emphasize the ability of EBOV to rapidly develop potentially adaptive mutations in multiple host species.

Influenza A virus (IAV) also has a high capacity to rapidly evolve and adapt to different hosts, not only through negative-strand RNA fragment re-assortment and recombination strategies but also due to its high mutation rate [183]. As mentioned above, many of these mutations can generate IAV subtypes that subvert host immune responses or affect viral tropism, particularly when they take place on the surface proteins hemagglutinin (HA) and neuraminidase (NA). For instance, the G186V mutation in HA is considered an adaptation of H7 avian flu to a subtype that can recognize human receptors [184,185]. The mutation R292K in the NA protein has been shown to promote resistance to oseltamivir, commonly used to treat influenza [186]. Other studies have shown that E627K substitution in the PB2 polymerase protein is responsible for increasing avian IAV host range, enabling viral replication in human respiratory epithelial cells [187].

Another recent example of virus mutation affecting human susceptibility involves SARS-CoV-2. Whereas most mutations can be expected to be neutral or mildly deleterious for the virus, a few mutations appear to confer a fitness advantage in certain contexts [188]. SARS-CoV-2 fitness-enhancing mutations can alter one or several aspects of virus biology such as transmission, antigenicity, infectivity or pathogenicity [170]. As the spike glycoprotein S mediates attachment to host cells and is the main target of host immune responses, mutations affecting the S protein are consequently of particular importance [189]. For example, the mutation N439K within the receptor-binding motif (RBM) of the S glycoprotein has been shown to enhance SARS-CoV-2 affinity for its cellular ACE2 receptor, and, additionally, has been found to render the virus less susceptible to neutralization by antibodies from recovered COVID-19 patients [190]. This is also the case with the glycoprotein mutation D614G, which has been shown to confer an advantage for infectivity and transmission [191]. It can also occur that several convergent mutations arise, probably during chronic SARS-CoV-2 infection or in reinfected or immunocompromised patients.

On the contrary, viruses that have evolved to be highly adapted to humans such as MeV, seem extremely stable at the genomic level. Taken together with its high R0, observations suggest that MeV is very highly optimized to suit humans as its natural and final host. However, this does not mean that viral evolution cannot be observed within the host itself. Indeed, when MeV reaches the CNS and provokes either measles inclusion-body encephalitis (MIBE) in immunodeficient patients or subacute sclerosing panencephalitis (SSPE), sequencing data frequently report mutations in the viral matrix, receptor-binding and fusion proteins [192,193]. These mutations generally abrogate the budding properties of the virus while often leading to a destabilization of the fusion complex and increased fusogenicity even in the absence of high-affinity entry receptors [72,192,194,195]. This hyperfusogenic phenotype seems more suitable for cell-to-cell dissemination rather than spread involving budding, a process that is potentially too toxic for the tissue. Among the common mutations, the study of the MeV F protein L454W mutation has helped in better understanding MeV evolution; indeed, this mutation was shown to be a hallmark of virus adaptation to brain tissue [195,196]. When used to infect mice intranasally, such mutants can still reach the CNS and kill the animal [194]. However, after only very few passages on Vero-hSLAM cells at 37 °C, in which the instability caused by the mutation is not required, and is even counterproductive, compensatory mutations such as E455G are able to re-stabilize the fusion protein and restore a wildtype phenotype [195,196]. Such observations tend to confirm that although instability allows MeV to enter numerous cells, even those devoid of high-affinity receptors, higher stability of the F protein is key to maintaining the circulation of the virus outside of the CNS, which may limit spillover to other species.

## 4. Host Factors Influencing Zoonotic Disease and Transmission

In terms of host factors, there are physiological, immunological and genetic factors in the host that, in combination with and depending on the infectious dose and route of exposure, can considerably affect the probability that an infection takes place. On a physiological level, there is a wide range of physical barriers that protect the host from being infected such as the skin, mucus, saliva, tears, stomach acid or the absence of receptors required by pathogens in certain tissues or cells [197]. The physiological barriers include the responses of the host that take place to remove the pathogen from the body, such as vomiting, coughing or sneezing. In addition, chemical and biological barriers contribute to eliminating the pathogen inside the host. For instance, the low pH in certain parts of the body or the release of defensins or lysozyme are examples of chemical barriers. Further biological barriers would include the microbiota that the host already contains and that compete with and prevent the colonization of the host by pathogenic microorganisms. Of note, these primary physical and chemical barriers work in parallel with the host cellular and humoral immune response to prevent infection. However, viruses have developed several strategies to evade host immune responses, from directly interacting and inhibiting host proteins to avoiding host immune surveillance or exploiting the immune system to their benefit [197], as partially illustrated in the preceding section.

The advances in genomics in recent decades have shown that host genetic factors in particular can severely affect certain infections; however, it is important to take into account that there is individual genetic heterogeneity in terms of response dynamics against infection and that this influences the risk to succumb to it and its severity [198]. Other than disease-independent parameters (e.g., sex, age, microbiome, contact rate), heterogeneity derived from disease-dependent parameters included infectivity, susceptibility and recovery rate amongst others. Importantly, these factors are complex and likely to be affected by various genes and environmental parameters [199,200].

Individual immune response heterogeneity plays an important role during infection since it will determine whether the host succeeds in battling the pathogen. Individual immune responses will also trigger different thresholds of immune activation and the intensity deployed against a particular pathogen [201]. The importance of individual variation is shown for instance by the existence of super-spreaders, individuals that disproportionately contribute to the high transmission of infectious agents [202]. Indeed, in the past, the dissemination of particular diseases or outbreaks have been shown to be perpetuated by super-spreaders; this is the case for MeV, the 2015 MERS and 2003 SARS outbreaks, the 2014–16 EBOV pandemic and, of course, more recently with SARS-CoV-2 and COVID-19 [6,203,204,205,206,207].

There are many factors that can perturb the immune system and generate variations in immune responsiveness between individuals. However, whereas immune proteins and populations are frequently quantified to determine variability, it is possible that such a complex system has compensatory pathways and strategies to achieve functional redundancy and maintain its functions. Amongst the factors affecting inter-individual immune variability, non-environmental factors such as age and sex seem to affect human immune cell and protein profiles [201,208]. It is believed that in older individuals, the immune system is characterized by the loss of certain immune cells such as monocytes and lymphocytes as well as a decrease in the diversity of T and B cell receptors, thus leading to repertoire perturbations that can affect immune response efficacy [209,210]. This is the case for instance with SARS-CoV-2 infection where elderly male subjects have been shown to have more severe outcomes and higher viral loads when compared to younger patients [211]. Another example of an age-related effect in the human antibody repertoire takes place in the context of influenza vaccination where elderly individuals have been shown to have a decreased diversity of expressed human antibody repertoire lineages [209]. Sex differences have also been shown to be present during influenza vaccination. Some studies have observed greater antibody responses to influenza vaccination in females than in males [212,213]. This could be due to differences in sex steroid hormones, which are known to affect the immune system; however, the specific reasons for this dimorphism are still unclear.

Another important factor influencing inter-individual variability includes host genetic factors which contribute to the composition and effector roles of specific immune system components. Apart from heritable medical conditions, there are non-heritable influences that can strongly affect the outcome of infection. For instance, the over- or under-expression of cellular receptors can severely affect the capacity of infection of pathogens that depend on these receptors. This is the case for the human immunodeficiency virus (HIV) co-receptor CCR5 gene. Individuals that present a homozygous 32-bp deletion in this gene prevent the virus from entering macrophages, thus conferring in many cases resistance to certain strains of HIV-1 [214,215].

Another example of individual heterogeneity would be the enhanced susceptibility to a pathogen due to underlying medical conditions. This has been shown to be the case for MERS and SARS-CoV-2 infections, where patients with pre-existing medical conditions that involve a decrease in immunity such as pneumonia or kidney diseases showed a higher severity of the disease and a worse outcome compared to patients with no pre-existing medical conditions [216,217]. Factors such as high blood pressure, a history of cardiovascular disease, obesity, chronic respiratory disease, diabetes, cirrhosis of the liver, splenectomy or even conditions such as schizophrenia can be added to this list [218]. Models have also suggested that immunocompromised individuals contribute to generating viral variants with pandemic potential by favoring the accumulation of mutations during virus replication and immune challenge [219].

These points are, however, not specific to emerging viruses. The fact that infection with a human virus such as RSV leads to a very variable response in humans suggests the presence of relevant differences in host factors. Several studies have shown that polymorphisms in innate immunity-related genes as well as various cytokines, surfactant protein D (SFTPD), Toll-like receptors 4 (TLR 4), inflammation gene myxovirus (influenza virus) resistance 1 (MX1) protein or vitamin D receptor (VDR) lead to genetic susceptibility to RSV bronchiolitis [220,221,222,223,224]. Thus, there are many genetic determinants for susceptibility and severity to RSV infection. Another example of the relevance of genetic variation includes the 2009 influenza pandemic (pH1N1), where genetic variation in immunity genes was associated with the severity of the disease. Examples of such polymorphisms are found in the CCR5 gene, the complement regulatory immunity gene CD55 or in interferon-induced transmembrane protein 3 (IFITM3), and all were identified as genetic markers for severe pH1N1 disease [225,226,227,228,229]. Certainly, the importance of IFITMs in particular in susceptibility to viral infections have been suggested by a number of studies [230,231,232].

However, some of these genetic associations are conflicting since they have not been replicated in other studies. These differences could be explained due to environmental factors. TLR4 genetic variability is an example of this controversy. Although in one study, TLR4 single-nucleotide polymorphisms (SNPs) were shown to be associated with the severity of disease during RSV infection [233], these results contrasted with a different study that showed no association of TLR4 genetic variability with RSV severity (but that did not include environmental exposure to lipopolysaccharide (LPS) in the study) [234]. Although microorganism influences contribute to shaping human immune responses, these differences highlight the importance of also considering the interactions between pathogens, environmental factors and a host’s genomic/transcriptomic landscape.

Faced with viral infections, hosts have developed strategies to detect and restrict viral replication. Many of these strategies involve the expression of host cell proteins known as restriction factors that are able to inhibit one or several steps of the viral life cycle thus limiting the impact of the infection [235]. As mentioned above, an important example of such host cell restriction factors is the human IFITM family that has been shown to mediate antiviral activity against many RNA viruses including IAV, HIV, West Nile virus (WNV), Zika virus or Dengue virus (DeV) [230,236,237]. Whereas some restriction factors, such as IFITM, affect viral entry and fusion, there are other host proteins such as myxovirus resistance (Mx) proteins that affect transcription, translation or protein synthesis of the virus. This is the case for hepatitis B virus (HBV) and HIV that are suggested to be inhibited by Mx protein through the formation of ring-like structures that sequester viral components thus preventing their replication [238,239]. Moreover, during MeV infection, the Mx protein has been shown to inhibit the virus in both the transcriptional and post-transcriptional phases of the viral replication cycle [240,241]. Some examples of host proteins that affect the late stage of the viral replication cycle include viperin and tetherin. In the case of viperin, this cellular protein can either localize in the plasma membrane of infected cells or co-localize in lipid droplets with viral proteins, in both cases disrupting viral budding. This phenomenon takes place during DeV, HIV and IAV infections [242,243]. Tetherin, on the other hand, prevents viral release by anchoring virus particles to the cell surface [244]. It has been shown that tetherin can exert a broad antiviral activity against several families of enveloped viruses such as retroviruses, filoviruses and arenaviruses [244,245,246,247,248].

Furthermore, when viruses infect cells, they cause changes in reaction to the infection. Besides immune responses and the expression of antiviral host proteins, it is important to consider the metabolic changes induced in infected cells [249]. Viral infections often change a cell’s metabolism by inducing higher glucose metabolism activity, or they can downmodulate one or more metabolic events [250]. For instance, certain viral infections induce metabolic reprogramming events that reverse an IFN response, which normally requires increased energy and lipid metabolism. Consequently, viral fusion is inhibited. This is the case of some flaviviruses, togaviruses, retroviruses, filoviruses, bunyaviruses and paramyxoviruses [251,252,253]. In this sense, nutrition has also been shown to influence the outcome of virus infections. For example, it has been shown that food deprivation makes neurogenic flu infection more severe; moreover, glucose supplementation has a protective role against lethal flu infection [254]. This effect is explained by an endoplasmic reticulum (ER) stress effect on the brain which causes more apoptosis in neuronal cells. There are also strategies that enhance host resistance by regulating the host’s metabolism by depriving cells of substrates required for pathogen viability. Indeed, the inhibition of glucose utilization in mice during HSV infection has been shown to diminish inflammatory lesions in the eye [255]. Thus, metabolic activities can influence the outcome of some viral infections.

The identification of efficient antiviral host cell restriction factors could play an important role in linking immune correlates of protection with the severity of infections for both human and animal viruses. The physiological relevance and the mechanism of action of the host responses associated with a specific or even several viral infections can elucidate important insights regarding the development of new therapeutics. Ideally, new treatments would target a broad range of viruses; however, this could be difficult due to the different mechanisms used between host cell subsets, inter-individual variability and the great diversity observed amongst viruses. Nevertheless, advances in our understanding of efficient host restriction factors remain important as they can facilitate the path towards the development of broad-spectrum antivirals.

Pre-existing immunity to a pathogen or to related pathogens will affect host susceptibility to disease. The nature of the pathogen and the amount of virus input received by a host over time will shape the composition and the responsiveness capacity against the same or related pathogens. For example, the presence of SARS-CoV-2 neutralizing antibodies and S-specific CD4+ T cells in individuals unexposed to the virus could be explained by the immunity generated due to previous coronaviruses [256]. Low-virulence viruses constantly infect and re-infect humans thus generating immune responses and shaping an individual’s immune system. This phenomenon can influence the risk of succumbing to infection at the individual and population levels.

In certain cases of emerging infectious disease, it is known that several rounds of stuttering chains of transmission are required before a pathogen can “successfully” emerge into a new host population. The initial, partial forays of the pathogen into the population may even serve to create some level of immunity and “prime” the conditions needed for/favoring more prolonged outbreaks following the reintroduction of the infectious agent [257]. This may be the case for the emergence of NiV in Malaysia where it has been suggested that the repeated exposure of pigs to infected reservoir bat species may have helped to create a herd immunity status supportive of virus circulation and spread [258]. For re-emerging human pathogens such as NiV that have already displayed a certain tendency for human-to-human airborne transmission, it is unknown if this phenomenon represents a factor influencing the human spread of the pathogen in populations that are likely regularly exposed to this or related viruses, for example, in areas of Bangladesh or India that are currently seeing fairly regular outbreaks of the virus, or even in populations that are potentially exposed to novel henipaviruses in Asia, Africa or South America [259,260,261].

Finally, specific environmental factors can affect the transmission and pathology of pathogens, particularly those that are airborne, including COVID-19. Air pollution, particularly exposure to microparticles and NO2 through inflammation of the respiratory tract and mucous membranes can directly favor infection [262,263,264]. In this sense, people living or working in cities or near major transport routes and hubs or in polluted atmospheres such as lorry drivers are particularly at risk. Air pollution can also indirectly favor the contraction of a disease and its severity, as microparticles reduce exposure to ultraviolet light and therefore the production of vitamin D and consequently can lead to a depletion of the immune system [265].

## 5. Discussion: Common Points, Knowledge Gaps and Lessons for the Future

It is evident that zoonotic spillover events represent major global health risks and important economic burdens, but the factors that determine such events are complex and remain poorly understood. The spillover of emerging animal pathogens to humans involves a highly dynamic combination of multiple processes that interact amongst themselves on different levels. Indeed, zoonotic spillovers are stochastic and require several transdisciplinary factors to take place, including initial transmission and infection and, if successful, efficient pathogen replication in the host leading to further, ongoing infection/transmission in the novel human host population.

**Figure 3:**
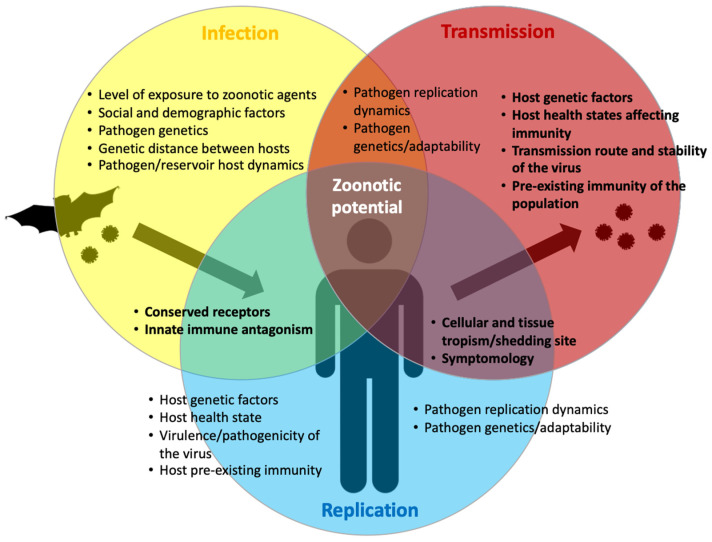
The major biological factors affecting the zoonotic potential of an animal virus to infect, replicate and spread efficiently in humans. Key factors are shown in bold. Common receptor usage and the ability of an emerging virus to inhibit innate immune responses in humans are major factors linked to spillover potential from an animal reservoir. A pathogen’s replication dynamics and genetic fluidity will affect its ability to adapt to a new host environment and immune responses. For ongoing human-to-human transmission following spillover, tissue tropism and disease severity/symptomology are important, as well as the ability of the virus to survive in the external environmental conditions required for transmission. On a population level, pre-existing immunity to infection will impact the ability of an emerging virus to spread and to adapt its fitness to humans as a novel host species. Any factors negatively affecting the host immune system will favor viral replication, adaptation and transmission. On a population level, this includes factors impacting general health states such as obesity, malnutrition, pollution and age demographics. Further details are given in the text.

Although the mechanisms involved are diverse, several common points can be identified in terms of pathogen–host cell interactions that would favor zoonotic transmission (Figure 3). Common receptor usage and the ability of an animal virus to inhibit innate immune responses in humans are major factors linked to spillover potential. As highlighted in this review, viral proteins that are able to antagonize host cell immunity factors could also be involved in the increased pathogenesis and virulence often seen in humans when compared to the virus’ natural reservoir species. In terms of ongoing human-to-human transmission following spillover, tissue tropism and disease severity/symptomology are important, as well as the ability of the virus to survive in whatever external environmental conditions are used for transmission. On a population level, any pre-existing immunity to infection will impact the ability of an emerging virus to spread and thus adapt to humans as a novel host species. Likewise, any impairment to a functioning immune system will favor viral replication, adaptation and transmission. On a population level, this translates to factors impacting general health states such as obesity, malnutrition, pollution and age demographics.

Viral cross-host exposures are most likely common occurrences but not all of them end up generating an outbreak [266]. Establishing the specific determinants of spillover for diverse pathogens and environmental/transmission conditions poses a complex challenge. To forestall new emerging viruses with epidemic potential the steps required to go from spillover to epidemics should be identified and studied. Finding functional links between a host’s species barriers to zoonotic infection and how the pathogen overcomes these will be crucial in helping to prevent future zoonotic spillover. Moreover, it is important to consider that spillover frequently involves the transfer of complex viral fitness landscapes to the host and/or between hosts and that the relative fitness of the emerging pathogen is often improved the more the virus is transmitted/replicates in the new host population.

Further detailed studies into the molecular, cellular and tissular pathogenesis mechanisms of human viral diseases and also of animal viral diseases with spillover potential are necessary to provide new clues to identify critical points that will be useful in assessing the transmissibility of future outbreaks of known or emerging/re-emerging pathogens. A number of studies have focused on estimating the number of viruses with zoonotic potential circulating in wild animal species [22,267] and even on developing or improving existing prediction models [3,268,269], but often, larger scale functional studies determining common correlates of zoonotic potential for putative emerging viruses for viral glycoproteins or innate immunity antagonism proteins, for example, are lacking.

Further investigation is needed to determine spillover patterns. It is important to be able to differentiate potential zoonotic spillover with the capacity to generate outbreaks or epidemics from background noise. Studies should involve transdisciplinary research involving ecology, epidemiology, virology, immunology, population demography and sociology, and on an international level. The conclusions of such studies can also be sharpened by merging computational and biological data [270,271]. Indeed, understanding and taking into account climatic, environmental, and ecological factors, according to the One Health concept, is also of paramount importance in predicting and preventing future spillovers. In the case of zoonotic bat viruses, for example, since spillovers and peaks of virus excretion from bats are often concomitant [272], it is crucial to decipher what drives and what can alter the pattern of virus excretion pulses. The bat breeding season seems to be associated with increased HeV and NiV shedding, because of a declining immunity level during this period. Despite most NiV outbreaks happening during the winter months in Bangladesh, serological survey suggests that NiV shedding by bats can occur at any time of the year and is cyclical rather than annual or seasonal [273,274,275]. Climatic factors were shown to determine HeV spillover patterns in horses, which could be linked to food shortage-related immune and behavioral changes in bats and horses [276]. Human activities also can create conditions that promote viral spillover, notably, when they contribute to depriving bats of their natural habitat which results in closer and more frequent bat-domesticated animals/livestock/human interactions and opportunistic feeding on anthropogenic food resources. Simple measures such as banning the planting of fruit trees adjacent to livestock enclosures, pigsties in particular, in order to avoid contact of domestic pigs with potentially infectious bat excretions, likely prevented further NiV outbreaks in Malaysia [277]. Anthropogenic pressures on land (deforestation, urbanization, etc.) and consequently on bats, leading to long-term changes in the composition and dynamics of bat populations and changes in seasonal movements, are thought to be drivers of future more regular spillovers of NiV, HeV and EBOV [278]. Climate anomalies (in terms of temperature or rainfall patterns for example) and climate change more generally likely drive the emergence and spillover opportunities of bat viruses by altering numerous aspects of bat physiology and ecology (foraging, stress induced by climate variability and related immune changes or migrations, etc.) [275,279]. In particular, food shortage and the altered body condition of bats are associated with decreased immunocompetence and HeV seroprevalence [280,281]. Overall, the ecological conditions experienced by wildlife reservoirs are thus key to predicting and shaping spillover occurrence and intensity [279].

Whereas some adaptation is often necessary for the efficient and prolonged transmission of an emerging virus within the human population, some animal viruses, including Ebolaviruses or NiV, appear to be already able not only to easily cross the species barriers between their host species and humans but also to be transmitted efficiently between humans [94,282]. On a biological level, one paper has estimated that more than 15,000 interspecies transmission events can be expected to occur between mammals alone by 2040 [283], and it has been estimated that some 10,000 circulating animal viruses, mostly RNA viruses, are transmissible to humans. In real terms, whether the outcome of an initial zoonotic transmission of a pathogenic virus to a potential human host is an isolated event, the onset of a localized outbreak or the onset of an epidemic or even pandemic is determined by the human-to-human transmission capacity of the virus as well as the socio-epidemiological factors that affect human behavior and health states, also including population density or age demographics or even geoclimatic variables [284,285]. Potentially, the key events that take place in such events can be identified or predicted. Indeed, not all spillover contributing factors can be controlled, but many can potentially be reduced. It is important therefore that risk assessment and prevention measures be taken to reduce spillover determinants.

Some of these actions include prevention measures and containment measures:

Prevention measures:

(i) Improve sanitary controls. This could be performed firstly by assuring basic health infrastructures to perform, in case of need, patient care, quarantines and diagnosis. Secondly, it would be important to undertake periodic sampling in farms and hotspots with high human–animal interactions. In order for it to be fast and efficient, surveillance should be genome-based thus allowing the early detection of emerging diseases. Of note, ideally, it is advisable to perform the sampling in wild animals, livestock and humans in contact with both. This would facilitate the early detection of the emergence of infectious diseases thus reducing the risk of spillover events;

(ii) It is crucial to reduce anthropogenic interference and urbanization in natural landscapes and forests. In addition to reducing biodiversity, deforestation frequently increases the contact between humans and wild animals since they look for new habitats close to humans, and it also favors the proliferation of vectors of potential human-associated diseases. This increases the risks of the emergence of infectious diseases;

(iii) Deploy resources to increase surveillance center infrastructure and human training in hotspots for emerging diseases for safe diagnosis and handling of potentially dangerous samples;

(iv) Identify and reduce potential biological, social and cultural factors that increase the risk of zoonotic pathogen transmission. There are several practices and behaviors that considerably increase the risk of spreading emergent diseases. Some of them include the consumption of contaminated aliments (fruits, palm sap, bushmeat…) and the cohabitation between humans and wild animals in rural areas with exposure to feces and urine. Reducing the consumption of potentially infectious foodstuffs and controlling the trade of wild animals would contribute to minimizing spillover risk. Moreover, the awareness of the potential diseases that wild animals could transmit would help to avoid cohabitation situations;

(v) Invest in research projects that aim to mitigate pathogens. This would include the generation of treatments and vaccines but also the identification of the mechanisms that render zoonotic viruses detrimental to humans and the host immune factors that can limit their infection and spread;

(vi) Regulate and inspect the biosafety protocols that need to be executed not only on a daily basis by all professional workers handling hazardous samples and animals (wildlife and livestock) but also protocols should be developed and applied and harmonized amongst countries in case of the emergence of infectious disease;

(vii) Develop preparedness strategies: it is relevant to increase society’s concern and responsibility and provide global public health education to the population. This can significantly reduce behaviors that increase the risk of spillover events [286]. This could also include better training for scientists and awareness in terms of communication strategies, particularly during an epidemic/pandemic with an aim of improving public relations and transparency concerning scientific reasoning and public health policy decisions.

Containment measures:

(i) There are several behaviors that can contribute to the amplification of an emerging disease. For instance, traditional burial practices that involve contact with potentially still infectious bodies or bodily fluids with no protection measures can be an important source of the spread of an emerging disease. Thus, regional cultural diversity can play a role in disease emergence prevention. Knowing the specific regional customs can allow the development of specific measures and public health practices that can limit transmission events. Another example would include the transmission dynamics that sometimes take place in high-density populated areas. During outbreaks, social contact should be reduced and large congregations of people should be avoided. Moreover, geographic human movements should be controlled to avoid the spreading of emerging diseases across borders, although this should be undertaken whilst always assuring the movement of critical supplies;

(ii) During a health emergency response, it is important to have coordinated work between health workers, governments, researchers and anthropologists. For this purpose, an appropriate expert committee should be responsible for assuring efficient and safe detection and response strategies and protocols that will have to be disclosed across the population. Communication is frequently disregarded during outbreaks, which contributes to continuous risk behaviors that amplify the pathogen through human-to-human transmission;

(iii) Need for blocking measures to adequately respond to emerging diseases. Rapid measures are essential to identify, understand and control the disease;

(iv) Readiness in the health system is also important in order to influence outbreak progression and control efforts. Having proper infrastructures, well-trained professionals in sufficient numbers, a good diagnostic system, standard barrier precautions and protocols and being able to assume the health and economic burden that emerging diseases bring is crucial in containing transmission and providing proper patient care. In light of the COVID-19 pandemic, it would appear that current international ‘stress’ tests for such a situation are either inadequate or inappropriate and require reevaluation [287,288,289].

In conclusion, it has been shown over the last few years, often dramatically, that known and unknown diseases can emerge in the human population due to contact with wild animals. These outbreaks, which in some cases become epidemics or even pandemics, can have a heavy medical, economic and social impact. Aside from continuing research studies into the biological mechanisms behind zoonotic spillover events and their amplitude, there are several actions that can contribute to preventing or reducing the impact of such events; however, in order for them to be efficient, these actions should be regularly applied. Although the emergence of zoonotic diseases cannot completely be avoided, it is possible to reduce the risk for it to happen and to introduce measures that will help to rapidly contain the disease after a spillover event. Strengthening these measures can help to ensure that future public health emergencies are better managed and contained.

## Data Availability

Not applicable.
